# Drivers of soil health across European Union – Data from the literature review

**DOI:** 10.1016/j.dib.2024.111064

**Published:** 2024-10-24

**Authors:** Shaswati Chowdhury, Maria von Post, Roger Roca Vallejo, Karen Naciph Mora, Jenni Hultman, Taina Pennanen, Antti-Jussi Lindroos, Katharina Helming

**Affiliations:** a Leibniz Centre for Agricultural Landscape Research (ZALF), Eberswalder Str. 84, Müncheberg 15374, Germany; b Department of Biology, Division of Biodiversity & Evolution, Lund University, Ecology building, Lund SE-223 62, Sweden; c ICLEI European Secretariat - Freiburg Office, Leopoldring 3, Freiburg 79098, Germany; d Sustainable Resources, Climate and Resilience, ICLEI European Secretariat - Freiburg Office, Leopoldring 3, Freiburg 79098, Germany; e Natural Resources Institute Finland (Luke), Latokartanonkaari 9, Helsinki 00790, Finland; f University for Sustainable Development in Eberswalde, Faculty of Landscape Management and Nature Conservation, Schicklerstr. 5, 16225 Eberswalde, Germany

**Keywords:** Meta-analysis, DPSIR, Land use, Soil health objectives, EU soil mission

## Abstract

Soil health in Europe has reached a critical point: it is estimated that 60-70% of European soils are unhealthy. Changes in land use, its intensity and the quality of management have significant impacts on soil health and soil related ecosystem services. A systems analysis of soil health dynamics requires an understanding of the drivers inducing changes in land use and management. The DPSIR framework was adapted to the context of soil health in the European Union (EU) and used as an analytical framework for identifying the drivers for soil health. A scoping literature review, divided in four parts based on different land use types (urban and industrial, agriculture, forest, and nature), was conducted using the PRISMA protocol. The identified drivers across all land uses have been adjusted and standardised in in-person and online workshops. This metadata set presents the typology of drivers sorted according to the EU soil mission's soil health objectives, land use type, and location. The literature review was conducted as part of SOLO (Soils for Europe), a EU´s Horizon Europe funded project and the dataset will support the co creation and knowledge developing platforms (think tanks) for each EU soil mission objectives.

Specifications Table

**Table:** 

Subject	Data article (Environmental Science)
Specific subject area	*Land use, soil health, agriculture, forestry, urban and industrial, nature, Policy and Law, Nature and Landscape Conservation*
Type of data	*Table (.xls files (dataset within tables), Images,* *raw, filtered, analysed*.
Data collection	A scoping literature review following the PRISMA protocol (https://www.prisma-statement.org/protocols) have been conducted during 15.06.2023 – 01.01.2024. The data collection took place in parallel for four land use types so certain standards regarding the timeline (2010-2023 and predictions up to 2100), the search engine (Scopus), language (English), and location (Regional to European level) have been set prior to the review process. Search strings were developed. The review was largely limited to the peer reviewed published literature available on Scopus but there were scopes outlined to enrich the search with grey literature. The data was compiled in an online Excel sheet on Google drive, with offline Endnote files for the sources. The list of drivers have been standardized across four land uses with an in person workshop (December 2023) and an online workshop (January 2024). Consolidation and partial sorting of the data took place offline using word, endnote and excel with a final organized list in excel for repository during November 2023 to April 2024.
Data source location	Data was acquired in multiple locations subjected to the correspondence of the authors. Source of the literature was mainly Scopus https://www.scopus.com/home.uri), supplemented by policy documents retrieved from Eur-Lex (https://eur-lex.europa.eu/)
Data accessibility	Repository name: Bonares Repository for soil research data Data identification number: (10.20387/zalf-a15w-f1kh) Direct URL to data: https://maps.bonares.de/mapapps/resources/apps/bonares/index.html?lang=en&mid=7a05f982-edd7-49e2-847b-afea02598959
Related research article	None.

## 1 Value of the Data

• The data supports the systems dynamics between drivers inducing land use change and management and soil health.
• The sorted data can contribute to identify the commonalities and differences of drivers of soil health across EU and these data sets can support future research on soil improving land use and management.
• The data can be further analysed to find gaps in research and establish research needs. Researchers can also replicate the studies to test the validity of the findings or modify the strategy to fit their needs.
• The data is collected using a systematic meta-analysis strategy hence the curated datasets provide an outlook on the drivers of soil and land use change and how it impacts the soil health in the European Union (EU).
• The data includes but is not limited to the extent of scientific studies on the topic in Scopus, as well as their corresponding metadata. The established meta-analysis strategy ensures the replicability and reproducibility of the process.

## 2 Background

Soil health in Europe has reached a critical point: it is estimated that 60-70% of European soils are unhealthy due to centuries of exploitation. Changes in the use, intensity or management of soil and land have a significant impact on soil health and thus, on the quality and quantity of ecosystem services soils can provide. While data and research are available about sol properties, little knowledge exists on the systematic understanding on drivers of soil health. The drivers induce changes in soil and land use management and are numerous and multi-faceted, including economic, social, institutional, and environmental factors. The primary aim of this article is threefold: i) to adapt DPSIR (drivers, pressures, state, impact, and response) framework to the context of changes in soil and land use and management and soil health; ii) to develop and employ a meta-analysis strategy based on the PRISMA framework for conducting a literature review to identify the drivers of future changes in soil and land management in the EU (European Union), and iii) to produce and communicate the metadata sets containing the results of the literature review. This data article builds the initial foundation for further exploration and analysis of understanding the future of soil health in the EU.

## 3 Data Description

The dataset contains bibliographic data compiled in an excel file containing three interconnected data sets. The first data set contains the final filtered set of studies after the literature review following the PRISMA protocol (for more details, see the following section). The contents of the table is described in Table 1.

**Table 1:** Description of the Data set 1.

Column		Description
A	Citation	Numeric as identifiers for references in column B
B	Reference	Detailed citation of references

The second dataset contains the finalized typology of drivers for future soil and land use. Table 2 elaborates the titles of the column and the description of the content.

**Table 2:** Description of the Data set 2.

Column		Description
A	Short Code	Shortened form of the category of drivers (described in Column C with the code letter in bold) and the numbers to identify a driver within a category
B	Name of the driver	Drivers for future soil and land use change identified and standardized across all land uses
C	Category	The name of one of the six categories (**D**emography, **E**conomy, **N**ature and environments, **P**olicy and institutional arrangements, **S**ocio-cultural context, and **T**echnology and Management) the drivers are divided (Column B) amongst.

The third dataset connects the previous two datasets and provides more information by connecting the drivers with different soil health objects (SHO) set by EU soil mission (cite) and subsequent locations. Table 3 provides the details of the dataset.

**Table 3:** Description of the Data set 3.

Column		Description
A	Short Code	See Table 1, Row 2.
B	Land use	Types of land use, can be one of the four, agriculture, forest, nature, and urban (shortened from ´Urban and industrial´) associated with column A drivers
C	Location	Location of the drivers associated with column A. Location is ideally limited to EU33 but some of the column contains values such as regions (e.g. Mediterranean) or other descriptive entities (e.g. drylands). More details in the following section.
D	Citation	Identifier numeric of the associated reference from Table 1. Column A.
E	SHO_All_of_them	Drivers (Column A), as well as their land use (Column B) and associated locations (Column C), that are to be taken into consideration to all soil health objectives
F	SHO_Soil_erosion	Drivers (Column A), as well as their land use (Column B) and associated locations (Column C), that are to be taken into consideration for soil erosion.
G	SHO_Land_degradation	Drivers (Column A), as well as their land use (Column B) and associated locations (Column C), that are to be taken into consideration for land degradation.
H	SHO_Soil_biodiversity	Drivers (Column A), as well as their land use (Column B) and associated locations (Column C), that are to be taken into consideration for soil biodiversity.
I	SHO_Soil_structure	Drivers (Column A), as well as their land use (Column B) and associated locations (Column C), that are to be taken into consideration for soil structure.
J	SHO_SOC	Drivers (Column A), as well as their land use (Column B) and associated locations (Column C), that are to be taken into consideration for Soil Organic Carbon (SOC).
K	SHO_Soil_literacy	Drivers (Column A), as well as their land use (Column B) and associated locations (Column C), that are to be taken into consideration for soil literacy.
L	SHO_EU_Global_footprint_on_soil	Drivers (Column A), as well as their land use (Column B) and associated locations (Column C), that are to be taken into consideration for sEU global footprint on soil.
M	SHO_Soil_pollution	Drivers (Column A), as well as their land use (Column B) and associated locations (Column C), that are to be taken into consideration for soil pollution.
N	SHO_Soil_sealing	Drivers (Column A), as well as their land use (Column B) and associated locations (Column C), that are to be taken into consideration for soil sealing.
O	SHO_Not_sure	Unsure what SHO the Drivers (Column A), as well as their land use (Column B) and associated locations (Column C), are associated with.

**Fig. 1:**
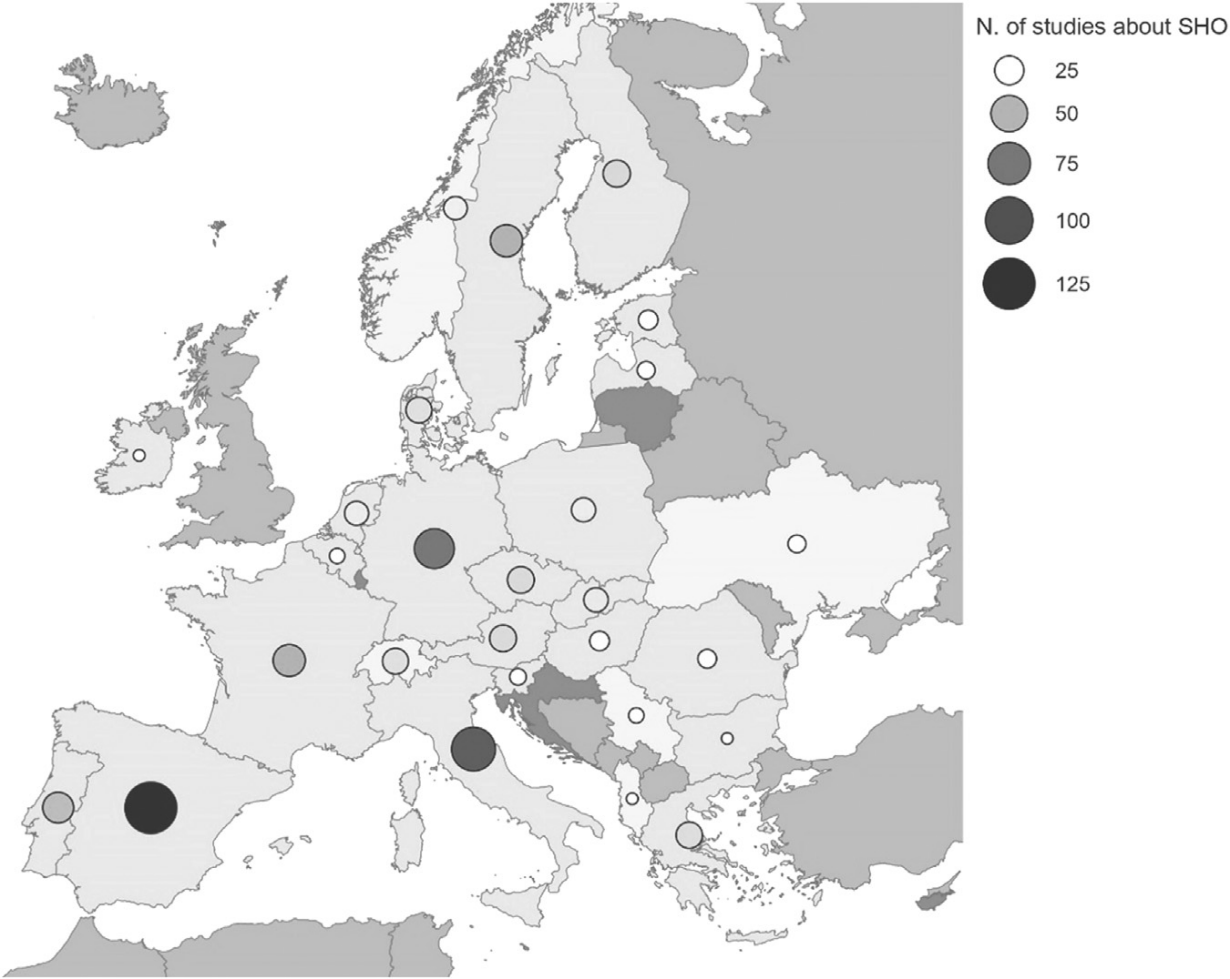
Geographical distribution and concentration of references.

**Fig. 2:**
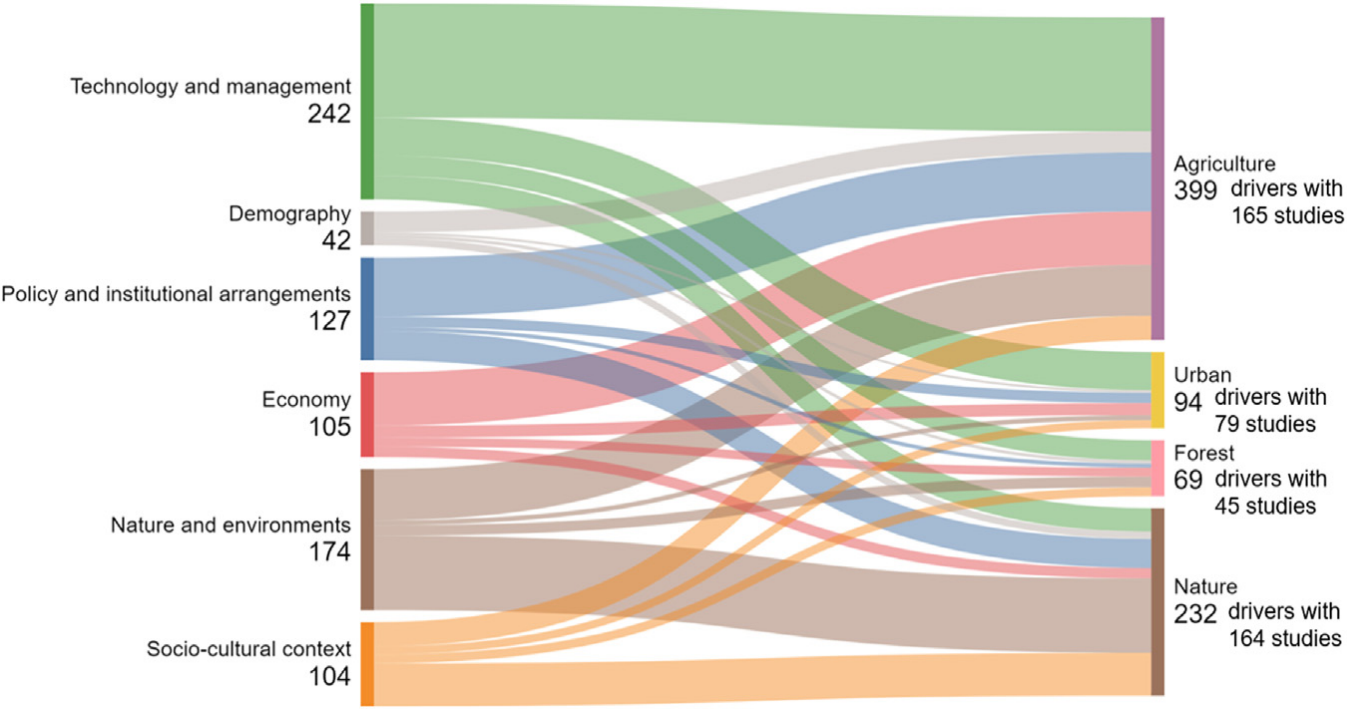
Distribution of drivers and selected studies in different driver's categories.

**Fig. 3:**
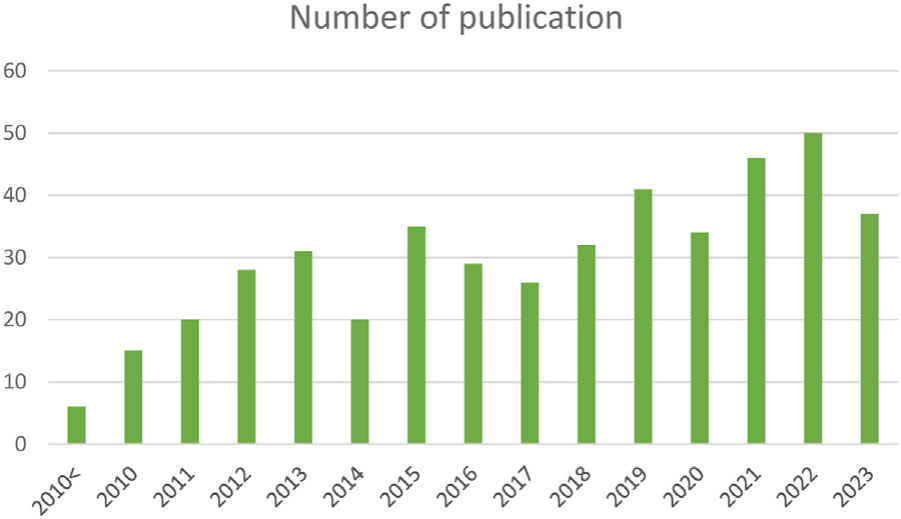
Annual distribution of all publications in the database.

**Fig. 4:**
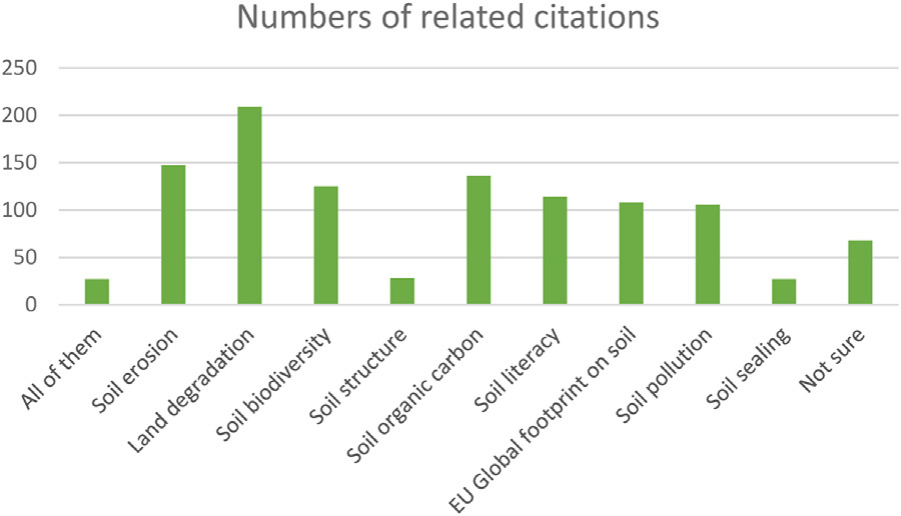
Distribution of citations in different Soil health objectives.

The following figures communicate the extent and diversity of the data. Fig. 1 presents the geographical distribution and concentration of references and Fig. 2 presents the distribution of drivers and studies in different drivers´ categories. In total, 125 drivers have been listed with 29 drivers in the Technology and management category, 12 drivers in nature and environment category, 12 drivers in the demography category, 43 drivers in the policy and institutional arrangement category, 12 drivers in the socio-cultural context category, and 17 drivers in the economy category (for details, Table 2 with the corresponding dataset 2). From the initial results, climate change has been identified across all land uses as a driver instigating multifaceted changes in land use and management and has impacts across all soil health objectives in all areas of EU. Urbanisation, changes in consumption pattern and demand, EU level directives, legislations as well as strategies, demand for renewable energy, are also identified as drivers with potentially significantly altering EU landscapes in future Fig. 3, Fig. 4.

## 4 Experimental Design, Materials and Methods

**Fig. 5:**
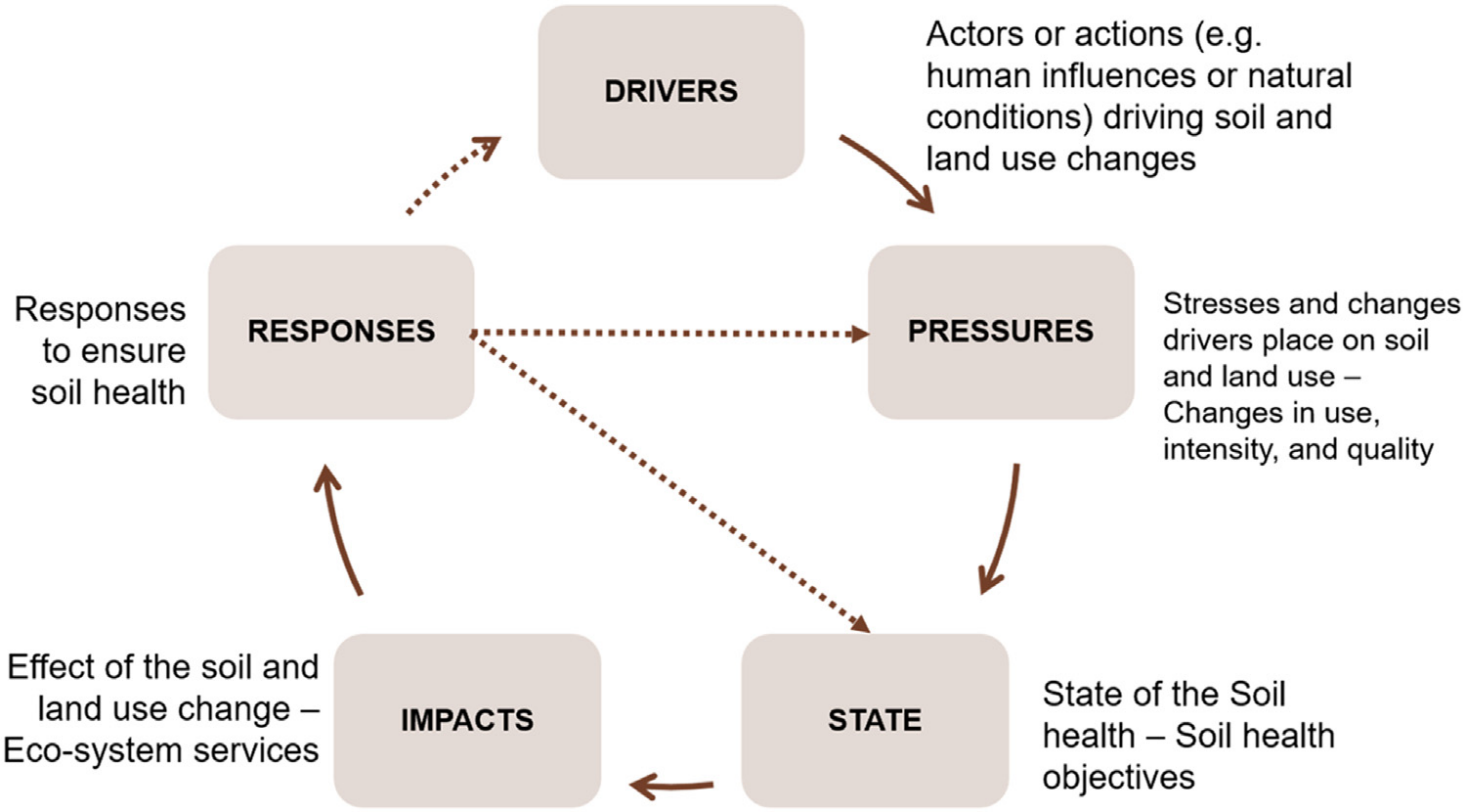
DPSIR Framework schematic for future soil and land use management for soil health (adapted from EEA [2]).

The DPSIR (Driving forces, Pressures, States, Impacts, and Responses) framework is an analytical tool for understanding the complex relationships between human activities and the environment [2,6,11] and has been widely adopted in different soil-focused national and European projects (BonaRes (https://www.bonares.de/), SMS (https://www.ecologic.eu/17696), PREPSOIL (https://prepsoil.eu/), SOLO (https://soils4europe.eu/)). The DPSIR framework (Fig. 5) was adapted to reflect the research aim of work package 3 of the SOLO project which is toidentify the drivers impacting future soil health across EU. In that regards, S- State of DPSIR is the state of the soil health, i.e. the soil health objectives (SHO) adapted to reflect the EU Soil mission objectives [1]. The I – Impacts in this case are the effects on soil and land based ecosystem services. The P-pressures are the changes that has or will take place in terms of soil and land use, management. The R - Responses are to be designed based on the inputs to ensure soil health and to target the pressures or the state. But the effective response design is done by addressing the root of the issue which in this case is the D - Drivers that causes the changes to take place.

**Fig. 6:**
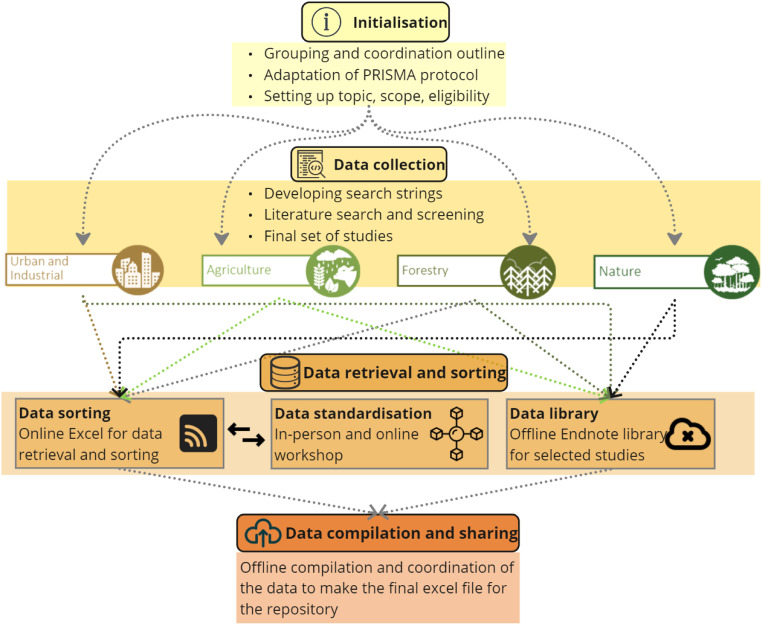
Meta-analysis strategy.

The meta-analysis strategy (Fig. 6) to identify these drivers impacting soil health was established following the PRISMA protocol [9] to guide and synchronise the process. The strategy consisted of four main elements. The first was ‘initialisation’ to start the process by setting up the working groups for the literature review, creating a coordination schedule, and setting up a general structure, scope and topic for the data collection. The second was ‘data collection’ to compile a series of studies accordingly, and to conduct a screening process by following a screening criteria, and to filterout the final set of studies to retrieve the data. The third was ‘data retrieval and sorting’ which consisted of strategies to compile, sort, and standardise the collected data. The final step was to assemble and compile the data collected in different datasets to create one file to upload to the repository. The elements for the meta-analysis strategy is discussed in detail below.

### 4.1 Initialisation

The metadata collection took place in four parts according to the four land uses; urban and industrial, agriculture, forest, and nature. The data collection was designed to take place in parallel for the four land uses by four different working groups, so certain standards regarding the timeline, the search engine, language and location had been set (Table 4) prior to the review process. The review was largely limited to the peer reviewed published literature available on Scopus but there were scopes outlined to enrich the search with grey literature.

**Table 4:** Details of data collection for the meta-analysis.

PUBLICATION TYPE:	PEER-REVIEWED, GREY LITERATURE (POLICY REPORTS, EU PUBLICATIONS, ETC)
TIME LINE	2010-2023 and predictions up to 2100
SEARCH ENGINES	Scopus
KEY WORDS	Select general and specific keywords related to land use types and drivers
LANGUAGE	English and local language (regional specific)
SPATIAL	Regional to European level

### 4.2 Data collection

**Fig. 7:**
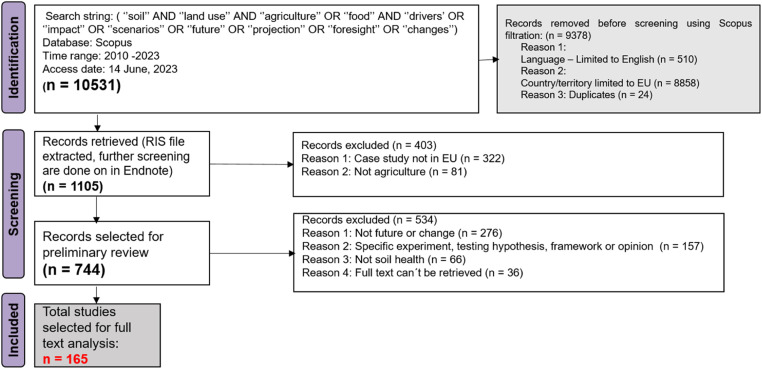
PRISMA flow diagram for literature review for Agriculture land use.

**Fig. 8:**
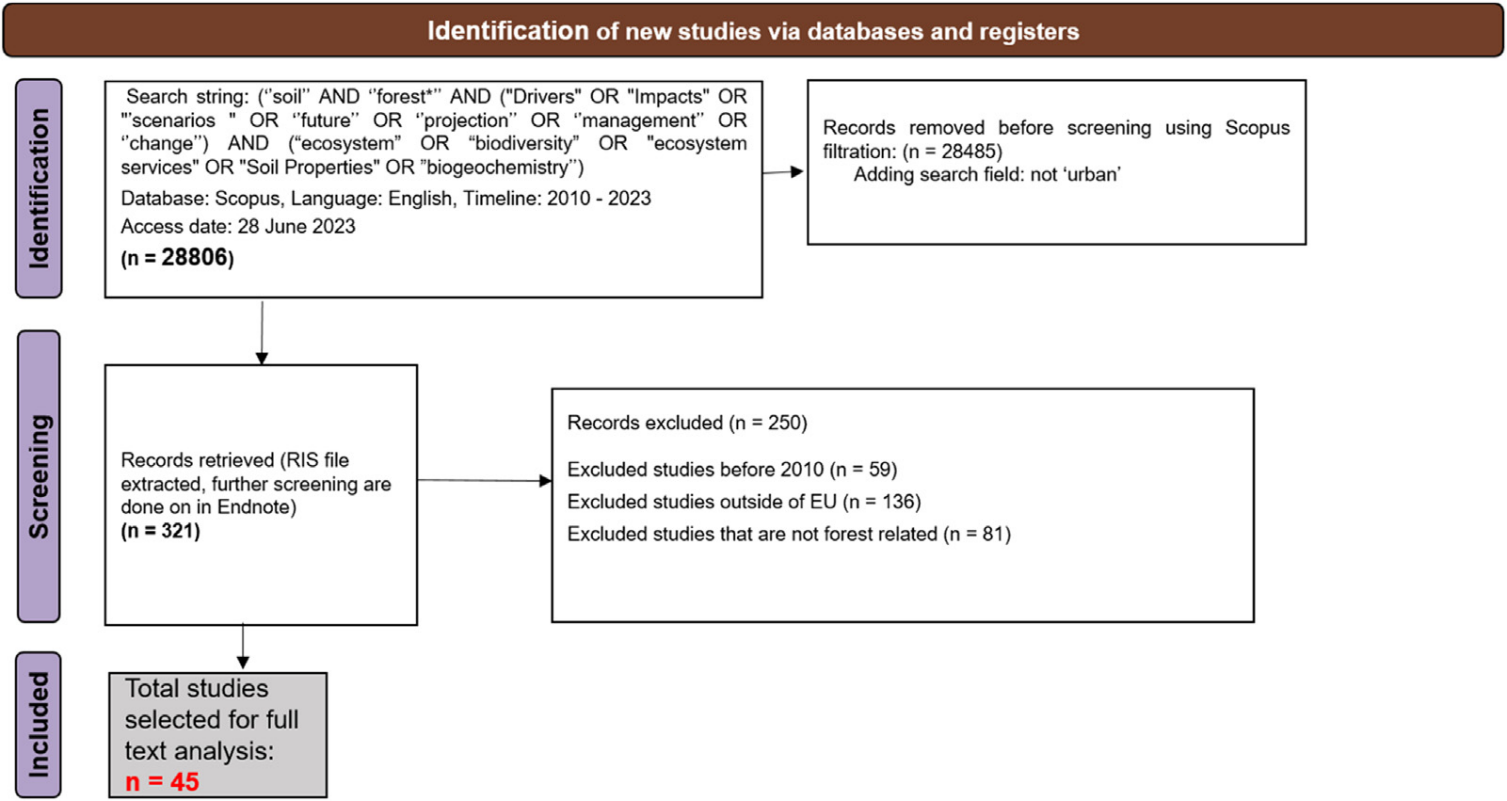
PRISMA flow diagram for literature review for Forest land use.

**Fig. 9:**
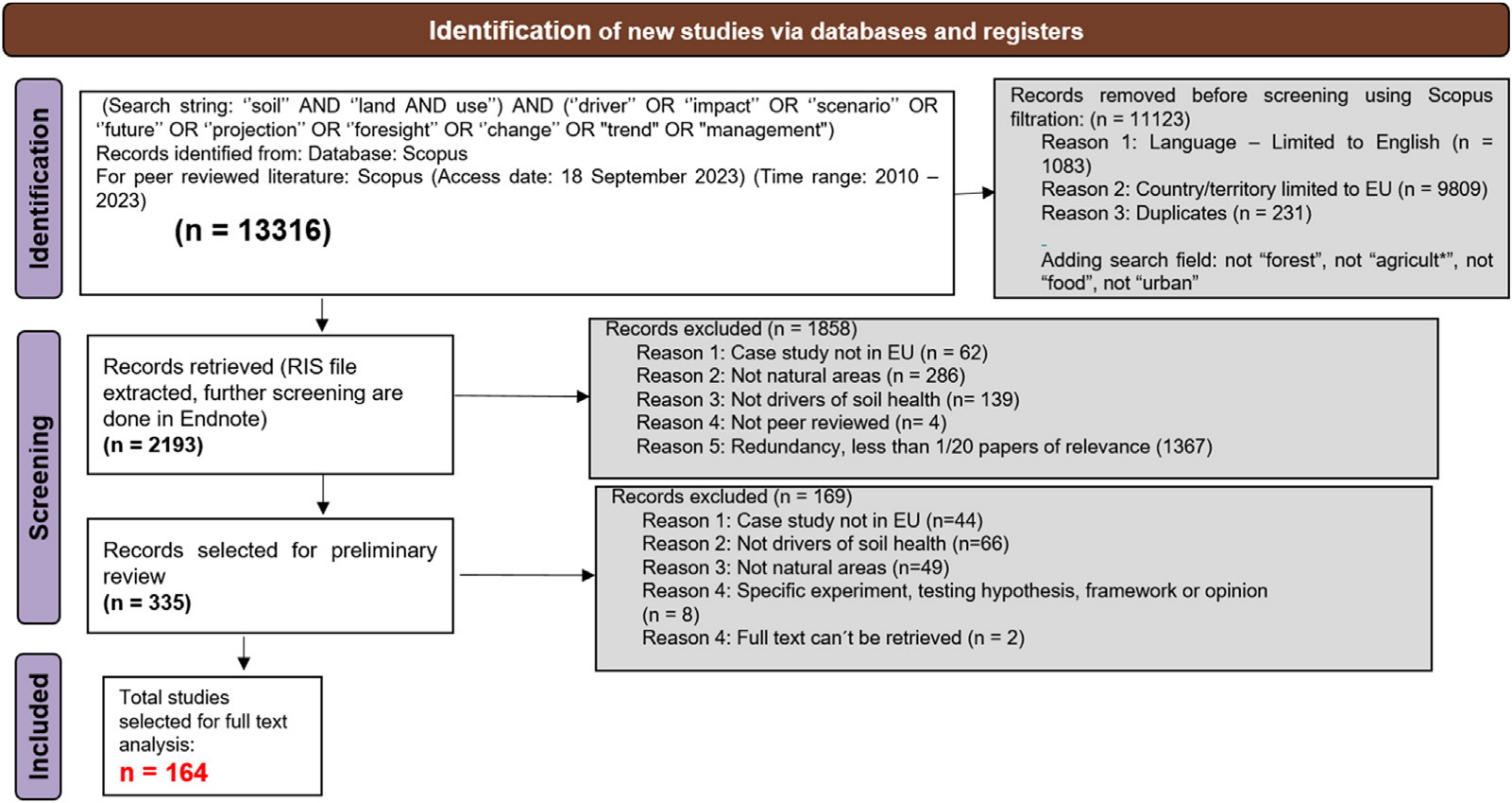
PRISMA flow diagram for literature review for Nature land use.

**Fig. 10:**
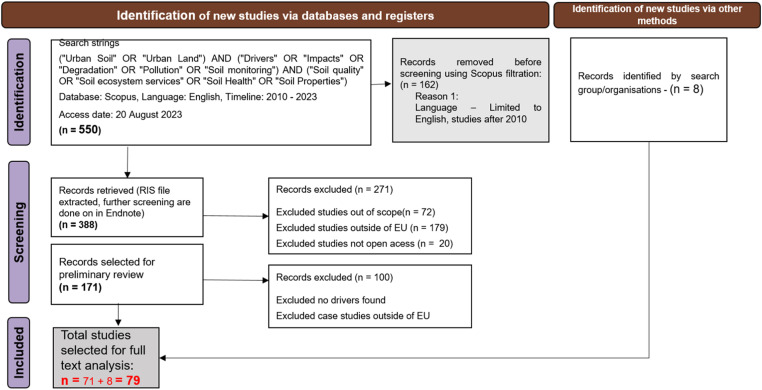
PRISMA flow diagram for literature review for Urban and industrial land use.

The data collection, as per the PRISMA protocol for the four land uses took place during the period between 15.06.2023 to 01.01.2024. Fig. 7, Fig. 8, Fig. 9, Fig. 10 summarises the PRISMA protocol carried out for the four land uses.

#### 4.2.1 Topic, scope and eligibility

Certain definitions were set up to guide the literature review. The drivers were defined according to their potential to motivate the following future changes:

• Changes in use – drivers of anticipated changes in land use compared to the present such as differences in type of use (uniformation, diversification, or gentrification), degree of use (intensification, extensification, or degradation), etc.
• Changes in management – drivers of anticipated changes in how the soil and land is managed compared to the present such as changes in regulation, practice, requirements, etc.
• Changes in management quality (smartness) - how well it may integrate multifunctionality and environmental, social and economic services, etc.

The scope of the literature as well as the drivers also included the soil health and soil quality, ideally associated but not limited to SHO. Details on the driver categories and how the SHO were defined can be found in the Data sorting section. Based on the scope, search strings were developed which were then used to find the initial set of literatures from the data base, Scopus.

#### 4.2.2 Screening

A large part of the literature was filtered out with the initial filtration criteria (see Table 4) and with the exclusion of duplicates,. Thereafter, the four working groups differentiated by the four land uses proceeded in slightly different ways. Most of the groups applied a next step of filtering the literature relevance based on reviewing the title and abstract. This step was usually done offline with an endnote citation list of the filtered literatures and the filtration focuses on more specific criteria such a location of case studies, if it is related to soil health or changes, or just a test of hypothesis. The screening criteria and process for all land uses are detailed in the Fig. 7, Fig. 8, Fig. 9, Fig. 10. The final selection of studies were analysed for their full text to fill up the sorting tables (4 and 5) in excel. In total, 53,203 literatures has been filtered among four land uses to end up with the final list of 451 studies Fig. 8, Fig. 9, Fig. 10.

### 4.3 Data Retrieval and Sorting

The data was mostly retrieved and collected online in an Excel sheet on Google drive, with offline Endnote files for the sources, during the period of 01.07.2023 to 01.03.2024. Consolidation and sorting were done mostly offline with a combination of the following: Word, Endnote and Excel, during 01.11.2023 – 31-04.2024. A compiled Endnote library was created to facilitate the development of the deliverable.

Due to the bulk and the complexity, the data is being processed in stages. The dataset presented in this paper is the summary of the data processed. It includes the typology of the drivers (second dataset) which are divided in six categories (see Table 2). The working groups were initially identifying and defining the individual drivers separately which were later standardised across all land uses. The data standardization took place in two workshops, one in person workshop, December, 2023, and another online workshop in January, 2024.

The selected studies were then sorted according to the standardised set of the drivers (i.e typology) and the associated SHO and the location identified (third dataset, see Table 3). The respective SHO definitions used for sorting is described in the Table 5. If a certain study could not be connected to a specific SHO, it was sorted to either two of the alternative categories, ´all of them´ or ´not sure (see Table 3).

**Table 5:** Definitions of the Soil health objectives (SHO) appropriated for the data sorting.

SHO	Appropriated definition for the meta-analysis
Land degradation	‘Degraded land in arid, semi-arid and dry sub-humid areas resulting from various factors, including climatic fluctuations and human activities’ [12]. also defined degraded land as ‘the result of human-induced actions which exploit land, causing its utility, biodiversity, soil fertility, and overall health to decline’. In the texts from the literature, several other concepts were also included when considering land degradation such as salinization, forest fires, aridity, drought, etc.
Soil organic carbon	‘Soil organic matter (SOM) is the portion of organic residues in soil in various stages of decay and the main component of SOM is carbon, also known as soil organic carbon (SOC)’ [5].
Soil sealing	‘Sealed soils can be defined as the destruction or covering of soils by buildings, constructions and layers of completely or partly impermeable artificial material (asphalt, concrete, etc.)’ [10].
Soil pollution	‘Introduction into or onto water, air, soil or other media of microorganisms, chemicals, toxic substances, wastes, wastewater or other pollutants in a concentration that makes the medium unfit for its next intended use’ [3]. Air pollution or emission have also been investigated but as a driver that induces soil pollution.
Soil erosion	‘The wearing away of the land surface by water, wind, ice, gravity or other natural or anthropogenic agents that abrade, detach and remove soil particles or rock material from one point on the earth's surface, for deposition elsewhere, including gravitational creep and so-called tillage erosion’ [4].
Soil structure	Soil structure is combined with soil biodiversity as an EU soil mission objective, but in this literature review soil structure and soil biodiversity were individually explored. Soil structure is defined as ´Arrangement of particles and organic matter to form aggregates which produce macro structures and micro structures in the soil´ [7].
Soil biodiversity	‘Variability among living organisms on the earth, including the variability within and between species, and within and between ecosystems’ [7].
EU global footprint on soil	‘EU's Ecological Footprint compared to that of the world’ [8]. EU global footprint on soil is not explored in the literature as phrased in the texts. But many associated concepts are explored in the literature as suggested by the definition, which can be attributed to this topic. The topics and concepts that are considered within its scope are for example, changes in demand, changes in dietary habits, increasing demand or production of renewable or bio-based energy or products, or any topics related to impact global greenhouse gas (GHG) emission.
Soil literacy	‘The state of knowing about or being familiar with soil. It concerns both a popular awareness about the importance of soil, and specialised and practice-oriented knowledge related to achieving soil health’ [1]. Soil literacy is a relatively less well explored topic as in, the phrase soil literacy has not been present in many references but many forms of it, as per the definition, has been explored in the literature for example, tools and models, systems, or methods for better understanding and procurement or transfer of soil related information and data.

### 4.4 Data Compilation and Sharing

The online collection of data across four land uses was finally combined and sorted offline in an excel file. An Endnote library consisting of all the filitered literature acroos all land uses was also compiled to support the dataset. The dataset was further structured following the guidelines of the respective online public repository (i.e Bonares) and was reviewed, and shared.

## Limitations

The literature review had been conducted searching a specific database and using specific sets of keywords. Other databases, specifically databases for grey literature, were not considered in this article which could potentially have resulted in a different and bigger set of study selection. The literature review was separately conducted across four land uses and the search keywords and filtration criteria were varied but still within the standards set by the data collection protocol.

## Ethics Statement

This data article is written in compliance with the ethical requirements for publication in Data in Brief. The dataset described in this article does not involve any human subjects, animal experiments, or data collected from social media platforms.

## CRediT Author Statement

**Shaswati Chowdhury:** Conceptualization, Methodology, Investigation, Data curation, Writing – original draft, Writing – review & editing, Formal analysis, Validation, Visualisation, Project administration. **Maria von Post:** Investigation, Data curation, Validation, Writing – review & editing. **Roger Roca Vallejo:** Investigation, Data curation, Validation, Project administration. **Karen Naciph Mora:** Investigation, Data curation, Validation, Project administration. **Jenni Hultman:** Investigation, Data curation, Validation, Project administration. **Taina Pennanen:** Investigation, Data curation, Validation. **Antti-Jussi Lindroos:** Investigation, Data curation, Validation. **Katharina Helming:** Conceptualization, Methodology, Writing – review & editing, Validation, Project administration, Funding acquisition.

## Data Availability

Bonares Repository for soil research dataMeta-analysis: Drivers of soil health across EU (Reference data).
